# The Recognition of Women in the Profession

**Published:** 1891-04

**Authors:** T. Dwight Stow

**Affiliations:** Mexico, N. Y.


### THE RECOGNITION OF WOMEN IN THE PROFESSION.

MEXICO, N. Y., March 7th, 1891.

EDITORS HOMŒOPATHIC PHYSICIAN :

I have just received and twice carefully read the "Charter, Constitution, and By-Laws of the Philadelphia Post-Graduate School of Homœopathics." All hail! the new birth. Thanks to the several gentlemen who have inaugurated the movement and presented the world with a *bona fide* institution for the promulgation of the principles and practice of pure Homœopathy. They built upon a firm foundation, and the superstructure should be equally firm. Yet I read with surprise in Section 3 of Article III, "No person shall be eligible to membership in this Association except a male citizen of the United States of America, of full age, of good moral character," etc.

Article III defines membership, but there is nothing in it to warrant the interpretation that the association is a "limited copartnership." Aside from the officials and faculty, it would seem well to enlarge the association by the annual election of any "of full age, of good moral character and habits, who shall subscribe to an undertaking to support and advance the principles declared in Article II of the Constitution." Why none but *males* are eligible to membership in the association will, to many, seem strange. Nearly seven-eighths of our patrons are women, and children dependent upon women; and the mothers, grandmothers, sisters, and female nurses of the land are the most able, persistent, and enthusiastic supporters of Homœopathy when they come to know the pure article. While women are so interested in supporting Homœopathy, while they are founding seminaries, hospitals, schools, asylums, churches, etc., and working day and night to maintain them, it does seem good policy to include the worthy of the sex in the membership of the association we take just pride in noticing to-day.

However, paradoxical as it may seem, in view of the fact that the letter and spirit of Section 3, aforesaid, creates a sort of close corporation "of male citizens," it is reassuring to see the name of a woman in the staff of lecturers. As we understand Sections 1 and 3 of Article III of said Constitution, females are totally excluded from honorary membership also.

There may be many good reasons why males can perform the duties of *active* membership better than females. But to exclude them from both active and honorary membership may turn out to be a "thorn in the flesh." For one, I am sorry that any association of persons aiming to advance the medical education of the times, should be handicapped by invidious distinctions of sex or class.

But all this sort of thing will, in the end, be of great benefit. For it forces a settlement of the vexed question, Who is a homœopathist? That once settled (as we think) in harmony with the Declaration of Principles, Article II of the before-mentioned Constitution, no person, male or female, subscribing to and *living* up to them, should be debarred membership in any association of homœopathists established for the *general* welfare.

I trust none will be unthankful for present blessings; yet we consistently may, and ought to seek more and better.

T. Dwight Stow.

